# Octreotide in Palliative Treatment of Hepatic Metastases: Is it Effective for Clinical Stabilization?

**DOI:** 10.4137/cmo.s409

**Published:** 2008-05-13

**Authors:** Kiki Pistevou-Gompaki, Nikos Eleftheriadis, Damianos Eleftheriadis, Christos Papaloukas, Apostolos Hatzitolios

**Affiliations:** 1 Department of Radiation Oncology, Aristotle’s University of Thessaloniki, Greece; 2 1st Propedeutic Department of Internal Medicine, Aristotle’s University of Thessaloniki, Greece

**Keywords:** octreotide LAR, liver metastases, palliative treatment

K Pistevou-Gombaki et al.^1^,^2^ presented their positive experience from the palliative use of octreotide LAR in hepatic metastases from non-neuroendocrine tumors. On the other hand, there are several negative prospective studies of octreotide efficacy in liver metastases and this policy is not accepted from al oncologists.^3^,^4^ According to this literature controversy and in continuation to the above-mentioned studies, we would like to report our recent experience from the efficient use of octreotide LAR in seven patients with symptomatic liver metastases from non-neuroendocrine tumors and in one female (80-years-old) with duodenal carcinoid.

Somatostatin has been shown to possess antimitotic activity against non-endocrine tumours, while octreotide, a somatostatin analogue, has shown considerable antitumor activity on animal models of various hepatic tumors. A possible antitumor mechanism of octreotide is a stimulatory effect on Kupffer cells, induction of apoptosis or other antiproliferative actions, which have been suggested but not proved.^5^,^6^ Furthermore, somatostatin and its long-acting analogues are effective in symptom control in patients with advanced neuroendocrine gastrointestinal tumours.^7^,^8^ Kouroumalis et al. reported a significant efficacy of octreotide for the management of hepatocellular carcinoma.^9^ Kapadia commenting on the improvement of quality of life after the administration of octreotide in patients with hepatocellular carcinoma, reported that the possible mechanism of this, should be the diminishing of the effects of various humoral agents and/or cytokines released from the tumor.^10^ However, the possible role of octreotide in palliative treatment of symptomatic liver metastases from non-neuroendocrine primary carcinomas remains controversial in the literature.

We report on seven patients, (3 females, 4 males), age: (63, 70, 71, 75, 72, 80, 81 years-old), with symptomatic liver metastases from different primary tumors-two from unknown primary origin, four from primary pancreatic carcinoma (two in the head and two in the tail of the pancreas) and one from duodenal carcinoid tumor-, who were palliatively treated by long acting octreotide IM (octreotide LAR) monthly, according to schema published previously.^2^

In all patients multiple liver metastases in both liver lobes were demonstrated, either by abdominal echosonography, computed-tomography (CT), magnetic resonance imaging (MRI) or a combination of them. In four patients pancreatic tumor in the head (two patients) and in the tail (two patients) of the pancreas was demonstrated. In two patients no primary tumor was found despite meticulous examination in general hospital with chest X-ray, abdominal echosonography, computed-tomography (CT), magnetic resonance imaging (MRI), gastroscopy, colonoscopy and urological examination. In one female, 80-years-old, a duodenal polypoid tumor, more than 5 cm, was found and histological examination was suitable of carcinoid tumor.

Clinical examination revealed slight enlarged liver in five patients and severe liver mass in two patients. All patients complained for general symptoms attributed to hepatic metastases, including right upper abdominal pain, loss of appetite, malaise and weight loss. At first instance all patients were palliatively treated by common analgesics and transdermal fentanyl, with slight improvement of the right upper abdominal pain. Eventually, when the liver pain deteriorated and produced severe morbidity, which could not respond to increase of the fentanyl doses, it was decided to treat all the patients with 20 mg long acting octreotide IM (octreotide LAR), once the first day, octreotide SC 0,5 mg X3/daily, days 2–14 and then 20mg long acting octreotide IM montly, as in our previous studies.^1^,^2^

The follow-up was done with clinicolaboratory examination and abdominal ultrasonography before and at one-month intervals after the initiation of octreotide therapy. The assessment of pain attributed to liver metastases was performed using the visual analogue scale (VAS) at baseline, at the time of octreotide IM injection and one week later (monthly). The improvement on palliative effect was also assessed every month using the analgesic intake scale (AIS) according to WHO (0 = no analgesics; 1 = NSAID; 2 = weak opioids; 3 = morphine).

An interesting point of our study was that all patients who had some degree of pain improvement, the local pain at the right upper abdomen relapsed when octreotide was temporarily stopped or at the end of every month just before the next injection of octreotide LAR and reduced again soon after the initiation of octreotide. One week after the administration of octreotide, the right upper abdominal pain was in general terms decreased. In fact, 2/7 (29%) patients reported excellent subjective improvement with more than 4 points decrease of VAS score. Moderate response (decrease of VAS by 2–4 points) was monitored in 2/7 (29%) patients, while only 3/7 (42%) patients showed slight relief of the pain (1 point decrease of VAS). During the first month of treatment, the AIS was significantly decreased from 2.8 ± 0.4 to 1.4 ± 0.5 (*p* = 0.023, Wilcoxon test). The data were analyzed using the Statistical Package for the Social Sciences (SPSS) for Windows (SPSS Inc., Chicago, 1993).

As far as the side effects are concerned octreotide was generally well-tolerated. Slight hyperglycemia was reported in 4/7 patients (57%), treated by specific diet. Decompensation of diabetes mellitus was monitored in 1/7 patients (14%) treated by insulin injections. No other severe side effects due to octreotide-treatment were reported. Only one patient discontinued the 3rd session of the regimen due to gastrointestinal side effects (nausea, diarrhea). During follow-up, six months after the initiation of octreotide therapy, local pain improvement was still reported in 4/7 patients (57%). One patient died three months after the initiation of treatment due to generalized malignant disease. Concerning the current status of subjects entered the study, 6 out of 7 patients (85.7%) are still alive, six months after baseline of octreotide-therapy. Totally 57% of patients showed some degree of pain relief, which could be attributed to octreotide use. The most important is that the values of VAS and AIS remained stable after the first month of improvement, as it is shown in Figure 1.

The major disadvantage of octreotide use is hyperglycemia and decompensation of diabetes mellitus, which may be significant but reversible.^9^ In the present, study although 71% of patients experienced hyperglycemia, this was not of clinical significance and none of the patients discontinued the therapy due to this side effect.

In this study we did not have control group and the diversity of the primary tumors, with the tumor-related morbidity, in relation to the placebo effect, which can result in improvement in cancer symptoms in as many as 40% of patients, made the evaluation of our results more difficult. However, taking into account the unpredictable behavior of end-stage malignant disease, the role of multiple parameters involved and the necessity of individualized policy in these debilitating patients, further studies are necessary in order to determine the subgroups of patients who might benefit from octreotide.

We consider the use of octreotide potentially effective in clinical stabilization and a good alternative in palliative treatment of symptomatic liver metastases in patients with end stage malignant disease. The positive results of the present study encourage further randomized controlled trials of octreotide in the treatment of metastatic liver disease from different non-neuroendocrine tumors.

## Figures and Tables

**Figure 1:**
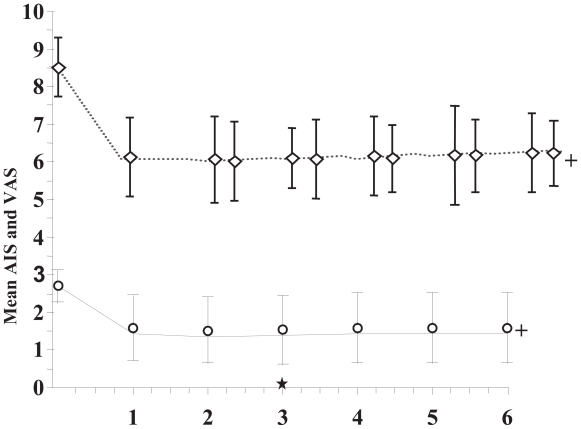
Time course of patients’ mean values AIS and VAS after octreotide administration.
